# Underwater noise mitigation in the Santa Barbara Channel through incentive-based vessel speed reduction

**DOI:** 10.1038/s41598-021-96506-1

**Published:** 2021-09-15

**Authors:** Vanessa M. ZoBell, Kaitlin E. Frasier, Jessica A. Morten, Sean P. Hastings, Lindsey E. Peavey Reeves, Sean M. Wiggins, John A. Hildebrand

**Affiliations:** 1 Scripps Institution of Oceanography, University of California San Diego, La Jolla, CA 92093 USA; 2 NOAA Channel Islands National Marine Sanctuary, Santa Barbara, CA 93106 USA; 3 Greater Farallones Association, San Francisco, CA 94129 USA; 4 NOAA Office of National Marine Sanctuaries, Silver Spring, MD 20910 USA

**Keywords:** Marine biology, Physical oceanography, Ocean sciences

## Abstract

Commercial shipping is the dominant source of low-frequency noise in the ocean. It has been shown that the noise radiated by an individual vessel depends upon the vessel’s speed. This study quantified the reduction in source levels (SLs) and sound exposure levels (SELs) for ships participating in two variations of a vessel speed reduction (VSR) program. SLs and SELs of individual ships participating in the program between 2014 and 2017 were statistically lower than non-participating ships (*p* < 0.001). In the 2018 fleet-based program, there were statistical differences between the SLs and SELs of fleets that participated with varying degrees of cooperation. Significant reductions in SL and SEL relied on cooperation of 25% or more in slowing vessel speed. This analysis highlights how slowing vessel speed to 10 knots or less is an effective method in reducing underwater noise emitted from commercial ships.

## Introduction

Low-frequency noise (5–400 Hz) in the ocean is dominated by commercial shipping^1,2^. In regions exposed to ship noise, ambient sound levels have risen over the past several decades due to increases in the number, gross tonnage, and horsepower of commercial vessels^3,4^. In addition to these parameters, vessel underwater radiated noise levels and vessel speed are positively correlated, suggesting noise pollution may be mitigated by reducing vessel speed^5–8^.

In 2014, the Channel Islands National Marine Sanctuary (CINMS) partnered with the Santa Barbara County Air Pollution Control District, Ventura County Air Pollution Control District, National Marine Sanctuary Foundation, and the Environmental Defense Center to implement a voluntary, incentive-based vessel speed reduction (VSR) initiative known as the Protecting Blue Whales and Blue Skies Program (hereafter VSR program)^9^. Enrollment was made available to companies operating container ships or vehicle carriers within the VSR zone, which extends approximately from Point Conception southeast to the Long Beach Harbor. Enrolled vessels were requested to reduce their speeds to a target speed to receive a financial reward. Vessels that participated in the VSR program from 2014 through 2017 received financial incentives of up to $2,500 per one-way transit and positive public press^9,10^. In 2018, the financial rewards ranged approximately from $1000 to $35,000 per company based on level of cooperation^11^. In addition to its original goals of reducing the risk of ship strikes on endangered whales and decreasing air pollution emissions, the VSR program also recognizes the opportunity to address underwater noise pollution in the Santa Barbara Channel (SBC). For example, acute and chronic noise pollution generated from commercial shipping has been documented to impact marine mammals, fish, and invertebrates in the form of acoustic communication masking, behavioral alterations, increased physiological stress, and reduced reproductive success^12,13^. Because of this, the potential for reducing noise pollution from commercial shipping by reducing vessel speed may allow the VSR program to address an even more comprehensive conservation initiative than originally anticipated.

**Figure 1:**
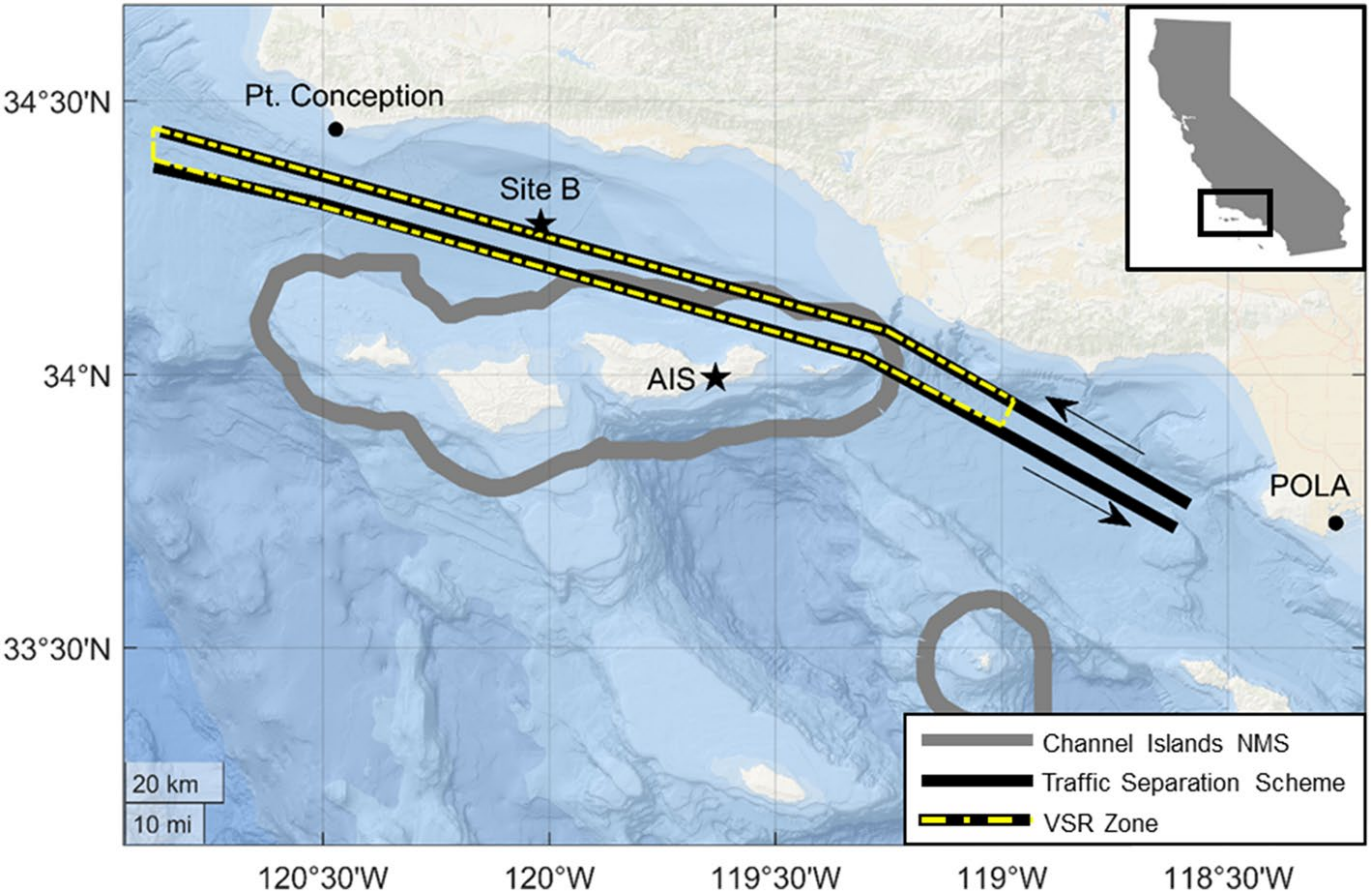
Map of the Santa Barbara Channel. The boundary of the Channel Islands National Marine Sanctuary (NMS) is shown as gray lines, the Traffic Separation Scheme is shown as black lines, and the Vessel Speed Reduction zone is shown as a yellow dashed line. Arrows denote the northbound and southbound shipping lanes. Stars show the acoustic recorder location at Site B and the Automatic Identification System (AIS) antenna location on Santa Cruz Island. Map tiles are courtesy of arcgisonline.com.

The SBC is an ideal region for the study of underwater noise pollution due to its position as a basin shielded from deep ocean noise by the presence of the Channel Islands and its proximity to the San Pedro Bay Port Complex (i.e., the ports of Los Angeles and Long Beach), which results in an abundance of low-frequency ambient noise that is directly correlated with commercial vessel traffic (Fig. 1)^14^. The Port of Los Angeles, in particular, is the busiest seaport in the United States, in terms of vessel traffic flow, and plays an essential role in the economic stability of California^15^. In addition to the economic importance of the SBC, measured by transported commerce, it is also a highly productive region that has led to a diversity and richness in zooplankton, fish, squid, and marine mammals^16–18^. The SBC is also an important summer foraging area for endangered baleen whale populations, as they aggregate in cold upwelling regions to feed primarily on krill^19–21^. Because of the ecologically important habitats in the SBC, noise pollution from commercial vessel traffic is a continuing management concern.

In this study, we evaluated the efficacy of a VSR program in the SBC for reducing underwater radiated noise of participating vessels. By using long-term acoustic records from the SBC, we compared ship source levels (SLs) and sound exposure levels (SELs) in relation to Automatic Identification System (AIS) speed over ground (SOG) values measured at the closest point of approach to the recording site. SLs are estimated with a surface-reflection compensated spherical spreading propagation model and compared between VSR participants at different SOGs and levels of cooperation. The analysis allows for a comprehensive acoustic analysis of two variations of the incentive-based VSR program (transit-by-transit and fleet-based). Both of the approaches showed a reduction in SL and SEL estimates for participating vessels compared to non-compliant vessels when fleets slowed at least 25% of their transits in the VSR zone.

## Results

From 2014 through 2018, paired AIS and acoustic recordings were extracted for 9297 vessel transits, including all types of vessels travelling through the SBC. The three vessel types under investigation in this study constituted 6738 of the extracted transits (72.5% of the AIS tracked vessels). Of these, 3778 vessel transits from 1299 unique vessels passed the 1 h isolation, no hydrophone cable strumming, and environmental conditions requirements for analysis inclusion. The transits were made up of 2677 container ships, 485 vehicle carriers, and 616 bulkers.

### Vessel speed

Across all ship types studied, the average SOG measured at the closest point of approach (CPA) was 7.3 ± 1.9 m s^−1^ (14.2 ± 3.6 knots, Table 1). The fastest ship type was container ships (7.6 ± 2.0 m s^−1^, 14.7 ± 3.8 knots, Table 1), while the slowest ship type was bulkers (6.1 ± 0.8 m s^−1^, 11.9 ± 1.6 knots, Table 1).

**Table 1:** Number of transits, mean (± standard deviation) speed over ground (SOG), broadband (5–1000 Hz) received level (RL), source level (SL), and sound exposure level (SEL) for container ships, bulkers, and vehicles carriers. The slope (regression coefficient) of the least-squares regression is shown for SOG vs. SL and SOG vs. SEL (SOG vs. SL|SOG vs. SEL) and the coefficient of determination (r^2^, SOG vs. SL|SOG vs. SEL) is displayed for three vessel types and their total.

Type	# of transits	SOG (m s^−1^)	RL (dB re 1 μPa^2^)	SL (dB re 1 μPa^2^ @ 1 m)	SEL (dB re 1 μPa^2^ s)	Slope (dB s m^−1^)	r^2^
All	3778	7.3 ± 1.9	110.0 ± 6.5	194.2 ± 6.6	158.1 ± 6.0	2.0|1.7	0.32|0.27
Container	2677	7.6 ± 2.0	110.3 ± 6.6	194.7 ± 6.8	158.4 ± 6.2	2.1|1.8	0.37|0.34
Vehicle Carrier	485	7.3 ± 1.6	108.5 ± 6.2	193.1 ± 5.7	156.8 ± 5.2	1.7|1.5	0.21|0.19
Bulker	616	6.1 ± 0.8	110.0 ± 6.2	193.0 ± 6.4	157.8 ± 6.0	2.6|2.2	0.11|0.09

### Broadband levels

Broadband (5–1000 Hz) RL (received level), SL, and SEL estimates from 3778 recorded transits of the three ship types studied are shown in Table 1. The distances that the broadband levels were measured at ranged from 600 to 4999 m. Including all ship types, average RL was 110.0 ± 6.5 dB re 1 μPa^2^ and average SL was 194.2 ± 6.6 dB re 1 μPa^2^ @ 1 m. SL estimates were highest for the container ships and lowest for the bulkers.

Broadband SEL allows for an estimate of total acoustic energy radiated into a region taking into account the transit duration. Including all ship types, the average broadband SEL was 158.1 ± 6.0 dB re 1 μPa^2^s. Average duration of 15 dB above background sound levels for all ship types transiting at different speeds was 727 s. Duration was specific to each ship transit and was dependent on speed and CPA. The average duration of transits with speeds less than 5.1 m s^−1^ (10 knots) was 676.2 s, while the average duration of transits with speeds greater than 9.3 m s^−1^ (18 knots) was 791.4 s. The duration of the faster transits was longer than the slower transits because of increased RL with increased speed, causing the RL to remain above the threshold for longer.

### SOG versus SL and SEL relationships

**Figure 2:**
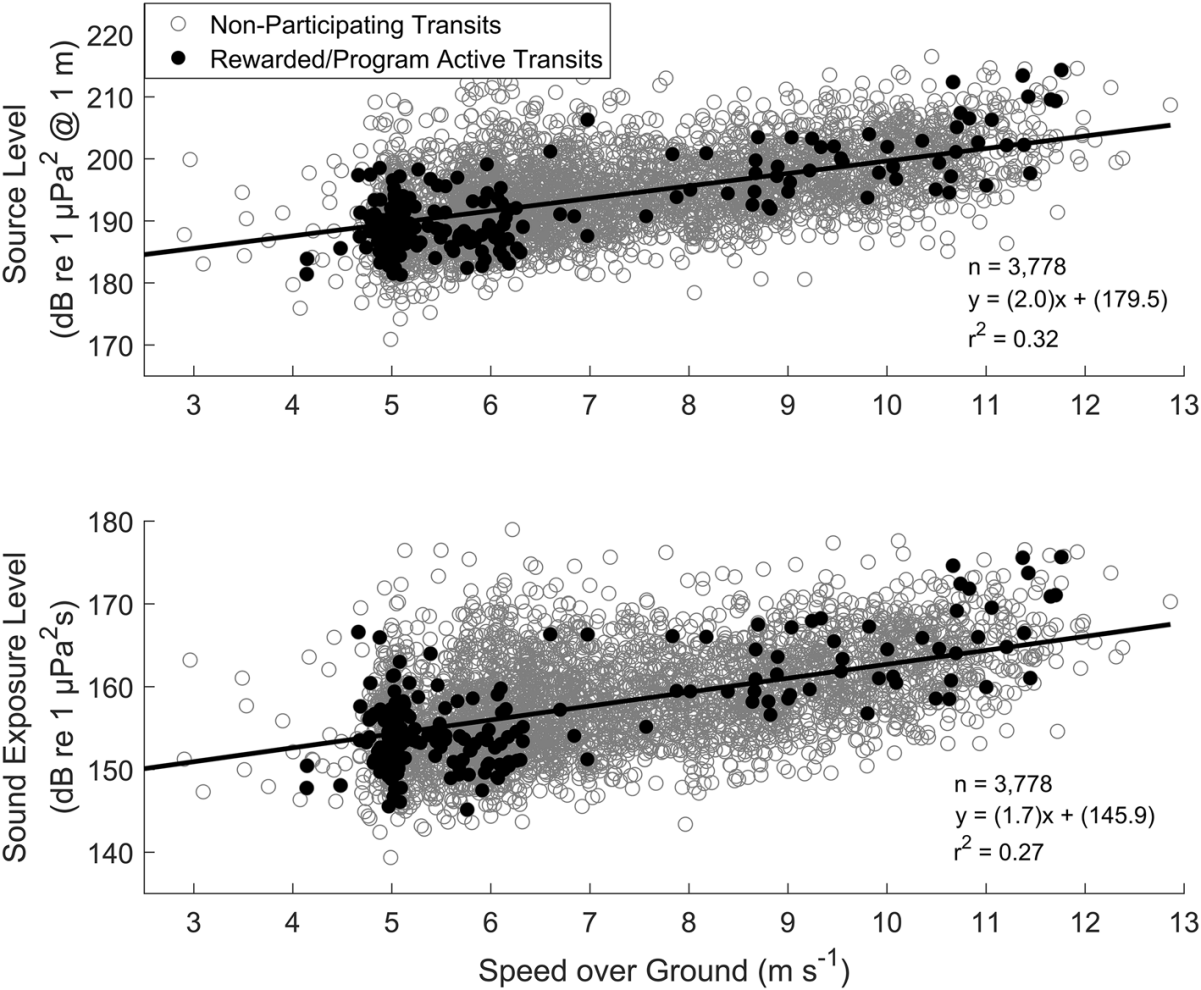
Broadband (5–1000 Hz) source level (dB re 1 μPa^2^ @ 1 m) and sound exposure level (dB re 1 μPa^2^s) in relation to speed over ground (m s^−1^) for 3778 cargo ship (container, bulker, and vehicle carriers) transits recorded between 2014 and 2018. Sound exposure level versus speed over ground shows a smaller positive slope than source level versus speed over ground. Rewarded transits from the transit-by-transit vessel speed reduction approach (2014–2017) and program active transits (all award tiers) from the fleet-based vessel speed reduction approach (2018) are shown in black.

The relationship between SOG versus broadband SL and SEL was determined by a linear regression model (Fig. 2). Including all three ship types, the slope of the linear least-squares fit between SOG and SL was 2.0 dB s m^−1^ (1.0 dB/knot, r^2^ = 0.32). Bulkers had the highest slope between SOG and SL (2.6 dB s m^−1^, r^2^ = 0.11), while vehicle carriers had the lowest slope (1.7 dB s m^−1^, r^2^ = 0.21) (Table 1). The slope of the linear least-squares fit between SOG and SEL for all vessel types was 1.7 dB s m^−1^ (0.9 dB/knot, r^2^ = 0.27), which was smaller than the slope of SOG and SL. Bulkers had the highest slope between SOG and SEL, although it was smaller than the SOG versus SL relationship (Table 1). The smallest slope reported was found between the SOG and SEL of the vehicle carrier transits (Table 1).

### Transit-by-transit vessel speed reduction approach

Of the 2,609 transits with paired acoustic and AIS data from 2014 through 2017, 152 transits (5.9% of transits) were associated with ships that participated in the transit-by-transit VSR program. The association was determined by matching the VSR program-supplied transit time and International Maritime Organization (IMO) identification number of the rewarded transits with the transits in the AIS and acoustic data. The 152 transits represented 47 unique vessels. The rewarded group consisted of 43 transits and the remaining 109 transits were recorded from the control group. Container ships represented 98.0% of the participating vessel transits, and vehicle carriers represented 2.0% of the participating vessel transits. Container ships represented 42 out of the 43 transits in the rewarded group, while 1 transit was a vehicle carrier. The control group consisted of 107 container ship transits and 2 vehicle carrier transits from 1 unique vehicle carrier.

**Figure 3:**
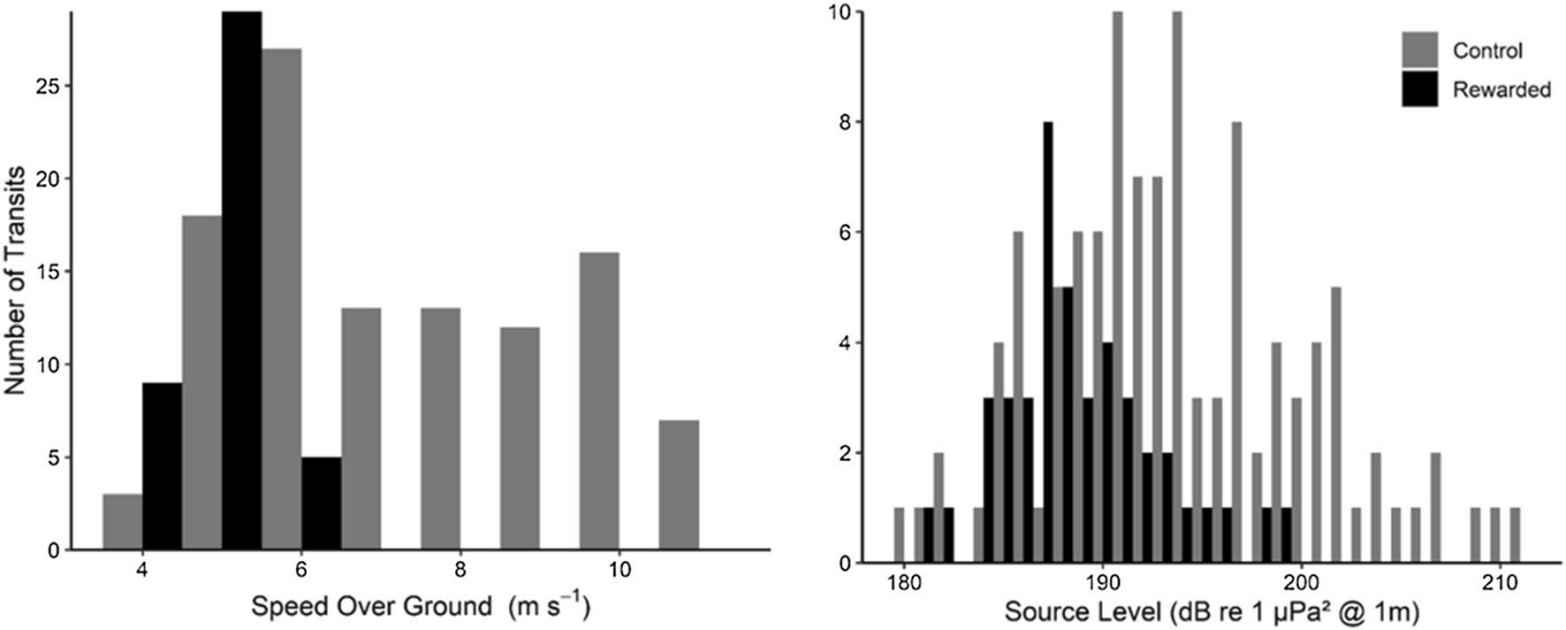
Histogram of speed over ground (m s^−1^, left panel) and broadband (5–1000 Hz) source level (dB re 1 μPa^2^ @ 1 m, right panel) for the control group and the rewarded group from the transit-by-transit vessel speed reduction approach (2014–2017).

**Figure 4:**
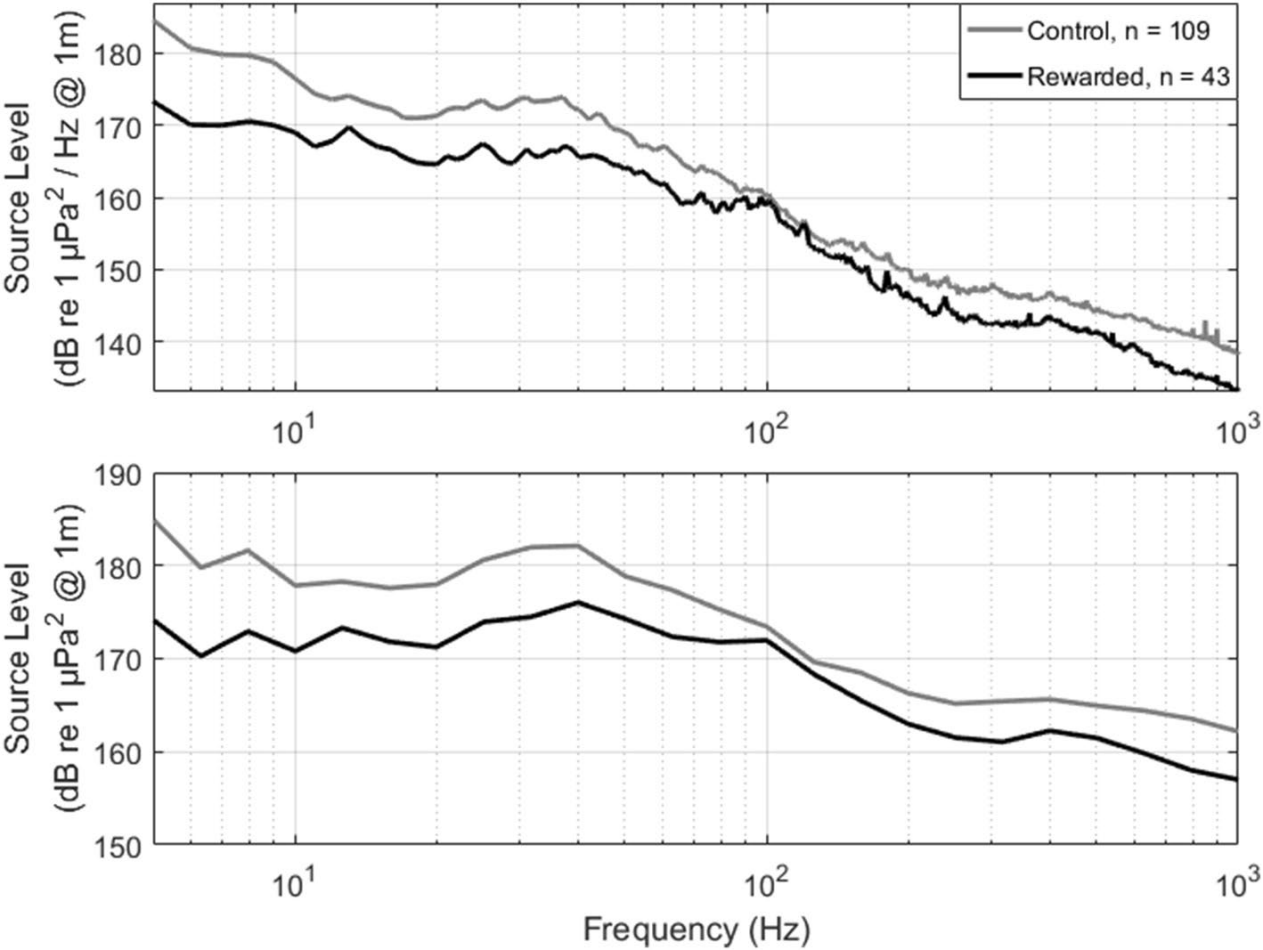
Mean source level spectra for control and rewarded groups during the transit-by-transit vessel speed reduction program in 1 Hz bins (top panel) and 1/3 octave bands (bottom panel).

The average speed over ground of the control group was 2.5 m s^−1^ faster than the average speed over ground of the rewarded group. The average SL of the control group was 5.2 dB higher than the average SL of the rewarded group (Table 1, Fig. 3). There was a less than 0.2 m difference in the effective source depth between the control and rewarded group. The reduction in frequency-dependent SL ranged from 0 to 10 dB depending on the frequency. The largest reduction (~ 5–10 dB) occurred at frequencies below 100 Hz (Fig. 4). There were lesser reductions above 100 Hz (~ 0–5 dB). The average speed over ground, broadband RL, SL, and SEL for the two groups are shown in Table 2. The histograms for SOG and SL (1 m s^−1^ and 1 dB bin, respectively) are shown in Fig. 3. The mean source level spectra for the rewarded and control groups are shown in Fig. 4.

**Table 2:** Number of transits, mean (± standard deviation) speed over ground (SOG), broadband (5–1000 Hz) received level (RL), source level (SL), and sound exposure level (SEL) for the transit-by-transit vessel speed reduction approach from 2014 to 2017.

Group	# of transits	SOG (m s^−1^)	Source depth (m)	RL (dB re 1 μPa^2^)	SL (dB re 1 μPa^2^ @ 1 m)	SEL (dB re 1 μPa^2^ s)
Control	109	7.9 ± 2.0	3.8 ± 1.0	109.6 ± 5.8	194.4 ± 6.5	157.1 ± 5.7
Rewarded	43	5.4 ± 0.4	3.6 ± 0.6	101.0 ± 3.7	189.2 ± 3.9	152.4 ± 3.4

### Fleet-based vessel speed reduction approach

In 2018, of the 1169 vessel transits that were recorded with paired acoustic and AIS data, 555 were from vessels participating in the fleet-based VSR program, of which 254 occurred when the program was active and 301 occurred when the program was inactive. Fleets were binned in award tiers based on percentage of cooperation. The participating vessel transits consisted of 50 recorded from fleets in the sapphire award tier, 113 from the gold award tier, 180 from the silver award tier, 177 from the bronze award tier, and 35 from the non-compliant tier (Table 3). Container ships made up 88.8% of the ship types in the transits recorded from the fleet-based program.

**Table 3:** Award tiers and percent cooperation for the fleet-based vessel speed reduction program in 2018. Number of transits, average speed over ground (SOG) measured at the closest point of approach, broadband (5–1000 Hz) received level (RL), source level (SL), and sound exposure level (SEL) are shown for groups program active and program inactive.

Award tiers (% Cooperation)	Program active					Program inactive				
	# of transits	SOG (m s^−1^)	RL (dB re 1 μPa^2^)	SL (dB re 1 μPa^2^ @1 m)	SEL (dB re 1 μPa^2^ s)	# of transits	SOG (m s^−1^)	RL (dB re 1 μPa^2^)	SL (dB re 1 μPa^2^ @1 m)	SEL (dB re 1 μPa^2^ s)
Sapphire (75–100%)	18	5.4 ± 1.2	109.9 ± 3.6	190.6 ± 5.0	156.1 ± 4.9	32	5.9 ± 1.3	114.9 ± 6.9	193.6 ± 7.9	159.6 ± 6.5
Gold (50–74%)	49	5.8 ± 1.4	108.3 ± 4.4	190.2 ± 5.1	155.7 ± 4.7	64	6.7 ± 1.6	114.5 ± 7.7	193.9 ± 7.9	159.8 ± 6.8
Silver (25–49%)	80	6.8 ± 2.3	110.6 ± 6.5	193.2 ± 8.1	157.5 ± 7.5	100	7.5 ± 2.1	116.7 ± 7.3	194.2 ± 7.9	159.5 ± 7.2
Bronze (10–24%)	89	7.8 ± 2.3	113.1 ± 6.1	195.6 ± 5.8	160.1 ± 5.7	88	7.7 ± 2.2	117.8 ± 7.7	196.4 ± 7.1	161.8 ± 6.2
Non-compliant (0–9%)	18	9.9 ± 1.8	115.7 ± 6.1	197.7 ± 6.3	161.4 ± 6.1	17	8.8 ± 2.1	118.6 ± 5.3	196.2 ± 7.6	161.1 ± 7.1

**Figure 5:**
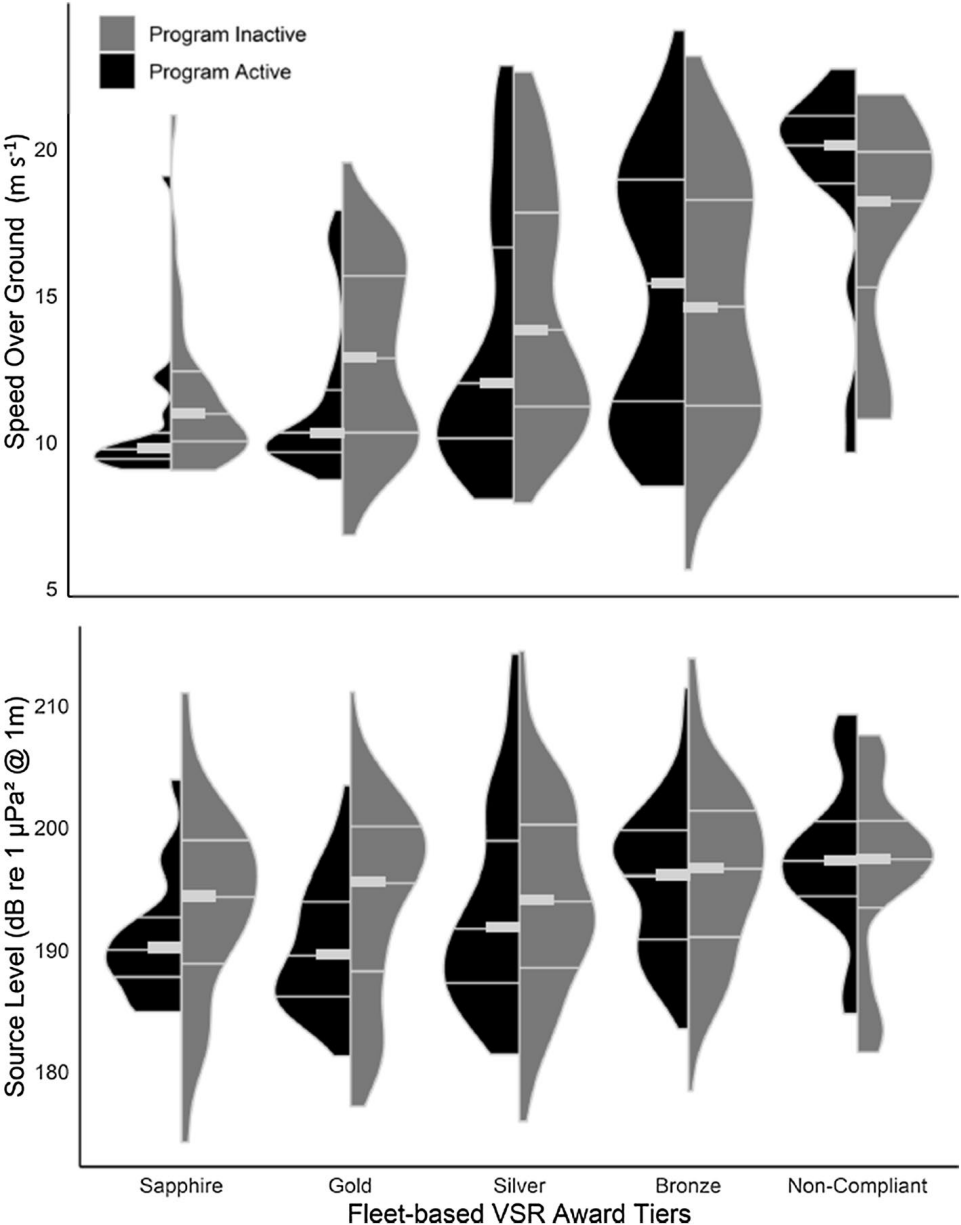
The distribution of speed over ground (m s^−1^, top panel) and broadband (5–1000 Hz) source level (dB re 1μPa^2^ @ 1 m, bottom panel) for each award tier while the fleet-based vessel speed reduction program (2018) was active and inactive. Quantiles (0.25, 0.5 and 0.75) are displayed on the distributions. The median is marked with a bolded line. Distributions were trimmed to the range of the data. All distributions were scaled to have the same maximum width.

The average SOG, broadband RL, SL, and SEL for each award tier are shown in Table 3. The distributions of SOG and broadband SL for transits in each award tier are shown in Fig. 5.

### Statistical analysis

In the transit-by-transit VSR program, there was a significant difference (*p* < 0.001) between the control and rewarded groups for SOG, broadband SL, and SEL. The average reduction in SOG, SL, and SEL between control and rewarded groups was 2.5 m s^−1^ (4.8 knots), 5.2 dB, and 4.7 dB, respectively.

While the fleet-based VSR program was active, broadband SL and SEL estimates from the sapphire, gold, and silver award tiers were significantly different from the non-compliant tier (Table 4). The greatest difference in broadband SL and SEL was between the gold award tier and non-compliant tier (7.4 dB and 5.7 dB, respectively). SOGs from the sapphire, gold, silver, and bronze award tiers were significantly different from the non-compliant tier while the program was active. The greatest difference in SOG while the program was active was between the sapphire award tier and non-compliant tier (4.5 m s^−1^).

**Table 4:**
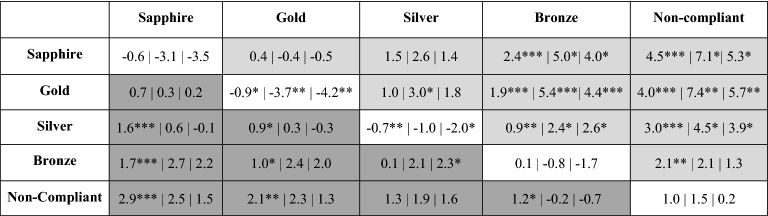
Matrix showing the difference in speed over ground, broadband (5–1000 Hz) source level, and sound exposure level (SOG (m s^−1^)|SL (dB re 1 μPa^2^ @1 m)|SEL (dB re 1 μPa^2^s)). Asterisks show degree of significance (*p* < 0.05 = *, *p* < 0.01 = **, *p* < 0.001 = ***). Light grey cells show the difference in means between award tiers while the fleet-based vessel speed reduction program was active. Dark grey cells show the difference in means between award tiers while the fleet-based vessel speed reduction program was inactive. White cells show the difference in means within an award tier while the fleet-based vessel speed reduction program was active v. inactive.

While the program was inactive, there were no statistical differences in broadband SLs between any of the award and non-compliant tiers. SOGs from the sapphire and gold award tiers were significantly different from the non-compliant tier. The greatest difference in SOG while the program was inactive was between the sapphire award tier and non-compliant tier (2.9 m s^−1^).

There was a statistical difference in SOG, SL, and SEL within the gold award tier (− 0.9 m s^−1^, − 3.7 dB, − 4.2), and a statistical difference in SOG and SEL within the silver award tier (− 0.7 m s^−1^, − 2.0 dB) while the program was active versus inactive. The SOG, SL, and SEL within the sapphire and bronze award tiers, and non-compliant tier were not significantly different while the program was active versus inactive.

All of the statistical results for the 2018 fleet-based approach are shown in the matrix in Table 4. The differences in means as well as the degree of significance (*p* < 0.05 = *, *p* < 0.01 = **, *p* < 0.001 = ***) within award tiers and between award tiers while the program was active versus inactive are displayed.

## Discussion

The broadband (5–1000 Hz) SL estimates in this study are consistent with the broadband SL estimate in Gassmann et al. (2017)^5^. The average SL for a ship transiting at 10.5 m s^−1^ (20.4 knots) in this study was 201.2 dB re 1 uPa^2^ @ 1 m, which is within 1 dB of the SL estimate for a ship transiting at the same speed at Site B in Gassmann et al. (2017). Our SL estimates are higher than SLs from other vessel noise studies at Site B because our SL estimates included a TL model which corrected for surface reflections that occur at sites with low inclination angles (Lloyd’s mirror) between ship near-surface sources and the seafloor-mounted acoustic recorder^7,8,22^. An approximate compensation to compare broadband source levels from 10 to 1000 Hz at Site B in McKenna et al. (2012) to Lloyd’s mirror corrected broadband levels from 5 to 1000 Hz can be derived by adding 20 to 27 dB^5^. Adding 20 dB to the average broadband SL estimates of containerships in McKenna et al. (2012) traveling on average at 10.85 m s^−1^ is 205.5 dB re 1 uPa^2^ @ 1 m, which is within 2–3 dB of the SL estimates of container ships traveling at the same speed in this study. When comparing overlapping frequencies, surface-reflection corrected mean SL spectra from vessels transiting on average at 5.4 m s^−1^ in this study were within 0–8 dB of the mean SL spectra calculated from a full-wave propagation loss model from vessels transiting at 5.8 m s^−1^ in Haro Strait, British Columbia^6^. Our SL spectra across frequencies was on average 3 dB higher than the SL estimates from Haro Strait, which may be due in part to the difference in depth at which the recording device was deployed^6^. The SL spectra from container and cargo ships estimated in St. Lawrence Seaway with a full-wave propagation loss model were within 5–9 dB of the SL spectra of container ships travelling at similar speeds in this study^23^, with our SL spectra across frequencies approximately 6 dB lower. Our surface-reflection corrected SL estimates from vessels transiting at 18 knots were within 0–10 dB from SL estimates from a single vessel transiting at 18 knots computed via Bayesian marginalization techniques^24^. Differences may be due to the high SL levels at prominent frequency tonals for the single ship spectra compared to our SL spectra averaged over multiple transits. The discrepancies between SL estimates measured at different locations, depths, and with varying propagation loss models is an ongoing issue and should continue to be investigated, in order to establish a method that enables comparison across sites and effective management plans for mitigating SLs. Because the transits in this study were recorded from the same site and the SL estimates were computed using the same transmission loss model, the measured change during the program active months is a reliable reduction.

Our results show a positive relationship between SOG and SL, similar to past studies^4,6,8,22,23,25^. Across all vessels, our relationship of 2.0 dB s m^−1^ (1.0 dB/knot) is within 0.2 dB s m^−1^ from the relationship found in Viers et al. (2016) and within 0.1 dB/knot from the relationship found in ships greater than 250 m in length in Simard et al. (2016)^22,23^. The relationship between SOG and SL for containerships in McKenna et al. (2013) was 1.1 dB/knot, which is within 0.1 dB/knot of the relationship for containerships in this study^8^. The relationship between SOG and SEL in this study had a smaller positive slope than the relationship between SOG and SL. As noted in McKenna et al. (2013), this is likely due to differences in the duration a vessel is transiting in the region^8^. Although the SEL slope is slightly less than the SL slope, we found that slower transits decreased the duration of time that the RL was above the 15 dB background sound level threshold in the majority of the vessel transits under investigation. The increased SL with increased speed may allow for some vessel sounds to travel farther distances and therefore be received above the threshold for longer durations.

Seasonal differences in radiated noise may also be contributing to differences in SL values. McKenna et al. (2013) identified that “month” was an important covariate in predicting SL estimates^8^. While our study incorporates a specific harmonic mean sound speed value in the transmission loss model for every month, there may be additional variability in underwater propagation that changes with season. For instance, there may be a decrease in radiated noise during the fall due to warm surface waters, creating downward refraction. In spring, the sound speed profile is closer to homogeneous, because of increases in storms creating a deeper mixed layer allowing the modified-Lloyd’s mirror model to be a better fit for spring environments, as sound travels in a straighter path than during the fall.

The Protecting Blue Whale and Blue Skies incentive-based VSR program was put into effect in the SBC in an effort to principally reduce air pollution impacts on local human populations and mitigate ship strikes on endangered whale species. At the inception of the program, reducing underwater noise from commercial shipping was regarded as a potential third conservation benefit of slowing large vessels. The added benefit of reducing underwater noise pollution from commercial shipping is quantified in this study. The transit-by-transit VSR approach (2014–2017) decreased SL estimates by over 5 dB and SEL estimates by over 4 dB for rewarded transits. The fleet-based approach (2018) allowed for significant reduction in SL and SEL estimates for ships that slowed 25% or more of their transits in the VSR zone (sapphire, gold, silver award tiers), when compared to non-compliant vessel transits. However, there was no statistical difference in SL or SEL between the bronze award tier and non-compliant tier, highlighting the limited reduction in noise levels for fleets not slowing down compared to the higher cooperating fleets. There was no significant difference between SOG or SL between the sapphire and gold award tiers, which may be due to the small sample size of the sapphire award tier. Additionally, the SOG of the sapphire and gold award tiers were significantly slower than the lesser cooperating fleets while the program was inactive, and there was no significant reduction in SOG within the sapphire award tier while the program was active versus inactive. This suggests that there may be fundamental differences between tiers that were not related to the Protecting Blue Whales and Blue Skies program. Additional voluntary speed reduction efforts exist in the Santa Barbara Channel seasonally and year-round, including voluntary VSR requests from NOAA—which run from May through November each year, the year-round Green Flag incentive program established by the Port of Long Beach, and the Vessel Speed Reduction Program established by the Port of Los Angeles^26–28^. Slow speeds observed from the fleets in the sapphire award tier while the Protecting Blue Whales and Blue Skies program was inactive may be due to fleets' involvement in these additional speed reduction programs throughout the year.

A vessel speed reduction program initiated in British Columbia also identified slowing vessel speed as an effective method to reduce source levels of commercial vessels^6^. The Enhancing Cetacean Habitat and Observation (ECHO) program’s average control speed was faster (9.7 m s^−1^, 18.9 knots) on average than the control speed in this study (7.9 m s^−1^, 15.4 knots). This allowed for an SOG reduction of 4.0 m s^−1^ (7.7 knots) during the ECHO program, compared to the Protecting Blue Whale and Blue Sky VSR program SOG reduction of 2.5 m s^−1^. Because of this, the ECHO program had a larger reduction in SL (11.17 dB for container ships) between the control and participating groups compared to the SL reduction found in this study. Similarly to this study, the ECHO program found the largest reduction in noise below 100 Hz, and the smallest reductions in the intermediate frequency range^6^. This is most likely attributed to the reduction in noise brought about by cavitation, which is most prominent in frequencies below 100 Hz.

An additional single ship transit in the Santa Barbara Channel can increase ambient noise levels averaged over a day by 1 dB^14^. Taking into account the average duration of approximately 12 min that a ship transit was 15 dB above background sound levels in this study, there would need to be at least 10 ships slowing down to half of their speed to result in a 0.7 dB reduction in daily average ambient noise levels. In order to maximize the reduction in daily average ambient noise levels, regulating the speed of vessels in the VSR zone, or fleets as a whole, may be necessary given the low cooperation with the incentive-based VSR program.

There are likely many reasons why certain fleets showed lower cooperation rates or would be less inclined to slow their speeds during the VSR program months; for example, scheduling, cost, competition, mechanics, weather and other issues along their overall routes. The incentive-based VSR program includes public recognition, which shows promise for the importance of positive public relations and its role in commercial shipping fleets’ willingness to participate in VSR programs^27^. Employing regulations for mandatory speed restrictions of 10 knots, as is done in the Seasonal Management Areas along the U.S. East Coast, would ensure that efforts to reduce the threat of lethal ship strikes, air pollution, and ocean noise are undertaken by all fleets in the region. Third-party certification and labeling programs such as Organic, Fair Trade, Rainforest Alliance, and Green Marine have promoted social and environmental sustainability, with appropriate private and public recognition^29,30^. Involving the VSR program and more commercial shipping fleets into certification programs like Green Marine may be an effective approach to further reduce speeds and improve voluntary cooperation or enhance government regulations. Additionally, the International Maritime Organization has identified quieting technologies, such as specific propeller and hull designs, as a potential method to reduce underwater noise. Retrofitting vessels in the commercial shipping industry may allow for a method to reduce noise on an international scale^31–33^.

Mitigating underwater noise generated from commercial shipping has the potential to reduce acoustic, physiological, and behavioral impacts that have been identified in marine mammals, fish, and invertebrates, allowing for an ecosystem-based approach to management. Effort into the investigation of biological responses to decreases in noise levels brought about by VSR programs is vital to ensure that measured SL and SEL reductions are adequate in reducing ship noise impacts on endangered whales and other marine organisms. Because the commercial shipping industry is a complex, intermodal system that operates under suites of constraints and externalities, an investigation into the various processes and stakeholders affected by the VSR program, including consumers, may aid in discovering how to permanently build conservation into commercial shipping.

## Methods

### Ship passages

Locations of all ships, including ships that were not participating in the VSR program, transiting in the SBC were tracked by an AIS receiver located on Santa Ynez Peak that is maintained by the Santa Barbara Wireless Foundation (Fig. 1). AIS messages were logged continuously by an on-site computer and decoded with the ShipPlotter program (ver. 12.4.6.5 COAA) for further analysis. To isolate ship transits on the northbound shipping lane, the monitoring area included transits within a 6 km radius of the acoustic recorder described below. Transits on the southbound shipping lane were excluded in order to minimize the ranges from the ships to the recording device. The ship name, IMO identification, type, speed over ground (SOG), draft, and position (latitude and longitude) were decoded from the AIS messages. The effect of surface currents on SOG was estimated to be less than 0.1 m s^−1^, from a moored Acoustic Doppler Current Profiler (ADCP) in the Santa Barbara Channel^34^. Additional information, such as ship length, was obtained from Lloyd’s Register of Ships^35^. The detailed vessel type was identified with the Marine Traffic online database^36^. Ship types were categorized into three groups: container ships (including reefers), bulkers (including bulk carriers, general cargo, wood chip carriers, timber carriers, and other cargo types), and vehicle carriers (including roll-on roll-offs). Tankers were not targeted for speed reduction in the SBC in the Protecting Blue Whales and Blue Skies incentive program from 2014 through 2018, and therefore were not included in this study.

Vessels transits were eliminated if another vessel transit occurred within the monitoring area within 1 h to ensure that each ship transit was acoustically isolated^8^. To assess the ANSI/ASA (2009) environmental condition requirements for underwater measurements of ship sounds, wind speed from the National Oceanic and Atmospheric Administration (NOAA) buoy (station 46053) near the acoustic recorder were checked and any transits that were associated with wind speeds greater than 10.28 m s^−1^ were discarded, as higher wind speed can increase ambient noise levels^37,38^.

### Acoustic recordings

High-frequency Acoustic Recording Packages (HARPs) have been maintained at a long-term acoustic monitoring station (Site B) in the SBC (34° 16.2′ N, 120° 1.8′ W) at ~ 580 m depth, 3 km off of the northbound shipping lane from 2007 to present^39^ (Fig. 1). HARP hydrophone electronics were calibrated at Scripps Institution of Oceanography and select full systems were calibrated at the U.S. Navy’s Transducer Evaluation Center facility in San Diego, California. Acoustic recordings were collected at a sampling rate of 200 kHz over 1508 days between January 2014 and December 2018. To reduce computational requirements, the recordings were decimated by a factor of 20 resulting in a sampling rate of 10 kHz. The data were lowpass filtered with an 8th order Chebyshev Type I IIR filter to prevent aliasing during decimation. The acoustic recordings were scanned for data quality, and transits that were contaminated with low-frequency hydrophone cable strumming were excluded.

The underwater radiated source level (SL) from an individual vessel was estimated from the received sound pressure level (RL) by accounting for the frequency-dependent transmission loss (TL) at the distance from the source to the receiver at the closest point of approach (CPA),

1 $$SL = RL + TL$$

### Received level (RL)

Received levels for each vessel transit were averaged over the data window period that equaled the time it took the ship to travel its length, as defined in ANSI/ASA (2009)^37^ (Eq. 1). Received levels were calculated for each ship passage by dividing the sound pressure time series into 1 s non-overlapping segments. For each 1 s interval, a fast Fourier transform (FFT) and Hanning window with FFT length of 10,000 samples and no overlap provided the power spectral density (PSD) in 1 Hz bins. Ten times the base-10 logarithm of the PSD in 1 Hz bins was used to convert to sound pressure received levels in decibels (dB) referenced to a unit pressure density (1 μPa^2^). The frequency-dependent hydrophone calibration was then applied to the PSDs to achieve RL in dB re 1 μPa^2^. To compute broadband RL values, hydrophone calibration-corrected RL levels in 1 Hz bins were converted to linear sound power spectra densities and summed across the 5–1000 Hz band, which is the approximate band for which ships are the principal source of noise within the SBC. The broadband RL values were then re-converted into dB re 1 μPa^2^.

### Transmission loss (TL)

To account for surface reflection interference (Lloyd’s mirror) that may reduce sound measurements at recording sites at much greater horizontal distances than the water depth, such as at Site B, we used a modified Lloyd’s mirror TL model, which was demonstrated to reproduce the sound levels received from ships transiting near their CPA in the northbound shipping lane^5,40^. A combination of the Lloyd’s mirror model and a spherical spreading model was used to account for the surface induced, source depth-dependent increase in TL seen with decreases in frequency^41^. The modified Lloyd’s mirror model utilizes the Lloyd’s mirror model from 5 Hz up to the frequency at which the Lloyd’s mirror TL and the spherical spreading model intersect. From the frequency of intersection to 1000 Hz, a spherical spreading model is used^5^. The intersection point between models depended on the source depth (see Effective Source Depth section) during a specific ship transit and ranged from 46 to 701 Hz.

Harmonic mean sound speed required for the TL model was calculated with data from the California Cooperative Oceanic Fisheries Investigations (CalCOFI) and California Underwater Glider Network^42^. Harmonic mean sound speed was calculated by dividing the total depth by the sum of the time it takes the sound to pass through each layer of constant sound speed. Depth, temperature, and salinity were measured near Site B (34° 15.150′ N, 119° 51.200′ W) on CalCOFI line 81.8 and station 46.9 four times per year and multiple times per year by the California Underwater Glider Network on line 80^43^. Between these two data sources, 30 sound speed profiles were measured from 2014 through 2018. Of the 49 months with paired acoustic and AIS data in this study, there were 30 months with corresponding sound speed profiles. For months that did not have a sound speed profile measurement, the sound speed profile measurement with the closest date was used.

### Effective source depth

Propeller diameter and draft are directly related to acoustic source depth, as cavitation occurs near the tip of the rotating propellers^44^. Constructive and destructive interference from surface reflections depends on the depth and frequency of the acoustic source, requiring that propeller dimensions and draft be determined for use with the modified Lloyd’s mirror model.

**Figure 6:**
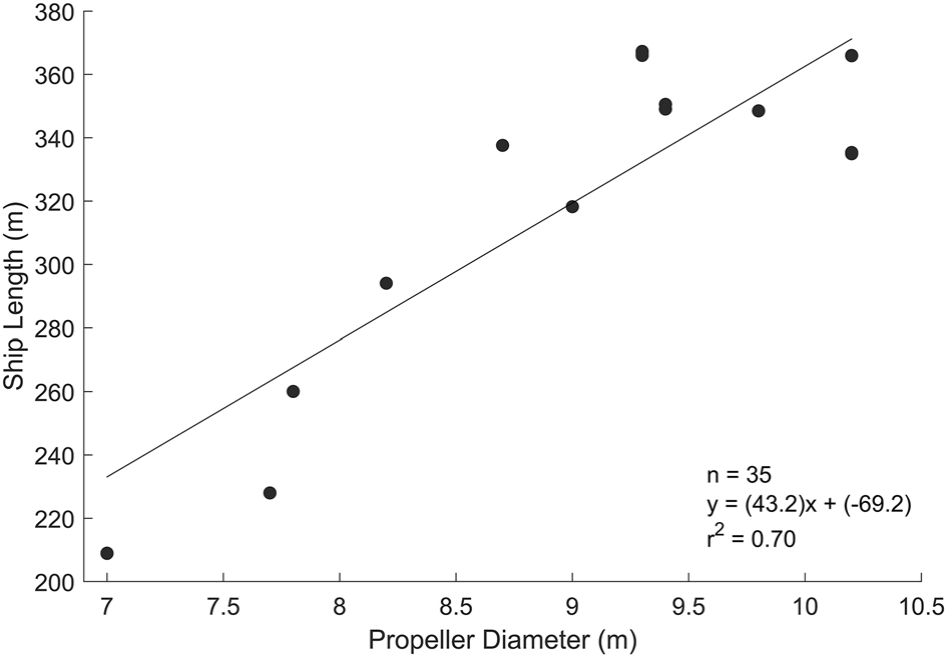
Ship length in relation to propeller diameter for a 35-vessel subset of the 2020 World Merchant Fleet. Many of the ships had nearly identical measurements, and are therefore overlapping.

Propeller diameters were modeled from a subset of 35 propeller measurements from the 2020 World’s Merchant Fleet utilizing the relationship between the propeller diameter and the ship length (Fig. 6). The depth of the acoustic source was assumed to be equal to 85% of the propeller diameter subtracted from the AIS reported ship draft^44^. Source depths ranged from 0.3 to 11.1 m with an average of 3.7 m ± 1.4 m.

### Source level (SL)

Source levels were estimated by adding the modified Lloyd’s mirror TL to the RL over the data window period. Broadband SLs were measured by summing across the 5–1000 Hz frequency band. Source level spectra were measured from 5 to 1000 Hz, and displayed in 1 Hz bins and 1/3 octave bands in compliance with ANSI/ASA (2009)^37^.

The relationship of SL versus vessel speed was determined by calculating the slope (regression coefficient) of the least-squares linear-fit, minimizing the sum of the squares of the deviations of the data from the model. The relationship was measured for each ship type.

### Sound exposure level (SEL)

The sound exposure level (SEL) was estimated as if each ship were transiting directly over the HARP by subtracting the frequency dependent TL from SL to estimate RL (in units of μPa^2^) at various ranges of interest (ROI), and integrating over the duration of the transit:

2 $$SEL = 10log_{10} \int\limits_{0}^{T} {\sum\nolimits_{fmin}^{fmax} {10^{ \wedge } \left( {\left( {SL\left( { f } \right) - TL\left( { f, t } \right)} \right) / 10} \right)dt} }$$

where T is the total transit duration in seconds. To keep the SEL measurements consistent with past ship noise studies in the SBC, T was defined as the time that the RL during the transit was 15 dB above background sound levels in the SBC, as ambient noise levels are elevated by approximately 15 dB when a ship is nearby^14^. ROIs for each transit ranged from the depth of the hydrophone to the distance the ship travelled over the duration T, before and after CPA. Estimated RLs were calculated for each 1 min interval over the duration of the passage.

### Vessel speed reduction approaches

The VSR program requested that enrolled vessels reduce speeds to a target of 6.17 m s^−1^ (12 knots) or less in 2014 and 2016 and 5.14 m s^−1^ (10 knots) or less in 2017 to present when transiting through the designated VSR zone. The target speeds were chosen because they have been shown to maximize reduction in ship strike risk, along with the added benefit of reduced air emissions^45,46^. The VSR program was active during the summer and fall (July 1 through November 15). During the winter and spring (November 16 through June 30) the VSR program was inactive, which served as control months to measure baseline noise measurements from the participating vessels.

In 2014, 2016, and 2017 the VSR program utilized a transit-by-transit approach for vessel enrollment. With this approach, enrolled companies signed up individual vessels and selected transits at the beginning of the program season to receive financial rewards and positive public relations for traveling at the reduced target speeds in the VSR zone.

In 2018, the VSR program changed to a fleet-based approach to incentivize slow speeds across all transits taking place in the VSR zone. In the fleet approach, container ship and vehicle carrier companies that cooperated in the program were rewarded based on the percentage of nautical miles that all vessels in their fleets traveled at 10 knots or less during the 2018 program season in the VSR zone. Companies with fleets that demonstrated higher percentages of cooperating transit miles were awarded with financial rewards and positive press. The award scale adopted by the VSR program in these years was based on percentage of cooperation (sapphire tier = 100–75%, gold = 50–74%, silver = 25–49%, bronze = 10–24%, and non-compliant = 0–9%).

Across all years of the program, historical AIS data was obtained from the United States Coast Guard, processed by the National Marine Fisheries Service, and provided to the VSR program administrators to monitor enrolled vessel speeds. In years that utilized a transit-by-transit approach, historical AIS data were analyzed to ensure the transits enrolled in the VSR program had an average speed of 6.17 m s^−1^ or higher prior to program enrollment. By doing this, the transit-by-transit VSR program was able to ensure that it was incentivizing companies that were voluntarily slowing transit speeds from previous years. The fleet-based VSR approach did not require a minimum historical average SOG qualifier for vessel enrollment in order to enroll all transits of involved fleets under operation by the participating companies. A total of 18.7% of the transits extracted with paired AIS and acoustic data, that met all required criteria for analysis, were associated with ships that participated in the VSR programs.

### Noise reduction statistical analysis

Transits of participating vessels in the Santa Barbara Channel from 2014 through 2018 were categorized into 4 different groups:

1. Rewarded (transit-by-transit approach)
2. Control (transit-by-transit approach)
3. Program active (fleet-based approach)
4. Program inactive (fleet-based approach)

The effects of the incentive-based VSR program were calculated for the transit-by-transit approach and the fleet-based approach separately. The effect of the transit-by-transit approach was calculated by comparing the rewarded group to the control group. A t-test was used to determine if the SL measurements of the two groups were equal or not equal. The null hypothesis for the t-test is that the SL measurements of the control group and the rewarded group were equal.

A Kruskal–Wallis test was conducted to determine the effect of the fleet-based approach by comparing the fleet award tiers to one another, and comparing each fleet award tier to itself while the program was active versus inactive. The null hypothesis of the Kruskal–Wallis test is that mean ranks of the SLs between award tiers and within award tiers while the program was active versus inactive are the same. A Dunn’s test of multiple comparisons was conducted to establish any significant differences within and between specific tiers. *p*-values were adjusted with the Benjamini and Hochberg (1995) method to control for false discoveries with multiple comparisons^47^.

## Data Availability

Automated Identification System (AIS) data from the Santa Barbara Wireless Foundation is publicly available at https://sbwireless.org/current/. Sound speed databases are publicly available at https://calcofi.org/data/195-available-data.html and https://spraydata.ucsd.edu/projects/CUGN/. The online database for detailed vessel information is publicly available at https://www.marinetraffic.com/en/ais/home/centerx:-12.0/centery:24.8/zoom:4.
